# Whole exome sequencing identified novel *CRB1* mutations in Chinese and Indian populations with autosomal recessive retinitis pigmentosa

**DOI:** 10.1038/srep33681

**Published:** 2016-09-27

**Authors:** Yin Yang, Yeming Yang, Lulin Huang, Yaru Zhai, Jie Li, Zhilin Jiang, Bo Gong, Hao Fang, Ramasamy Kim, Zhenglin Yang, Periasamy Sundaresan, Xianjun Zhu, Yu Zhou

**Affiliations:** 1 Sichuan Provincial Key Laboratory for Human Disease Gene Study, School of Medicine, Sichuan Academy of Medical Sciences & Sichuan Provincial People’s Hospital, University of Electronic Science and Technology of China, Chengdu, China; 2 Department of Ophthalmology, Sichuan Academy of Medical Sciences and Sichuan Provincial People’s Hospital, Chengdu, Sichuan, China; 3 Department of Laboratory Medicine, Sichuan Academy of Medical Sciences and Sichuan Provincial People’s Hospital, Chengdu, Sichuan 610072, China; 4 Institute of Laboratory Animal Sciences, Sichuan Academy of Medical Sciences and Sichuan Provincial People’s Hospital, Chengdu, Sichuan, China; 5 Key Laboratory for NeuroInformation of Ministry of Education and Medicine Information Center, School of Medicine, University of Electronic Science and Technology of China, Chengdu, Sichuan, China; 6 Retina-vitreous services, Aravind Eye Hospital, Madurai, Tamilnadu, India; 7 Department of Genetics, Aravind Medical Research Foundation, Aravind Eye Hospital, Madurai, Tamilnadu, India; 8 Chinese Academy of Sciences Sichuan Translational Medicine Research Hospital, Chengdu, Sichuan, China

## Abstract

Retinitis pigmentosa (RP) is a leading cause of inherited blindness characterized by progressive degeneration of the retinal photoreceptor cells. This study aims to identify genetic mutations in a Chinese family RP-2236, an Indian family RP-IC-90 and 100 sporadic Indian individuals with autosomal recessive RP (arRP). Whole exome sequencing was performed on the index patients of RP-2236, RP-IC-90 and all of the 100 sporadic Indian patients. Direct Sanger sequencing was used to validate the mutations identified. Four novel mutations and one reported mutation in the crumbs homolog 1 (*CRB1*) gene, which has been known to cause severe retinal dystrophies, were identified. A novel homozygous splicing mutation c.2129-1G>C was found in the three patients In family RP-2236. A homozygous point mutation p.R664C was found in RP-IC-90. A novel homozygous mutation p.G1310C was identified in patient I-44, while novel compound heterozygous mutations p.N629D and p.A593T were found in patient I-7. All mutations described above were not present in the 1000 normal controls. In conclusion, we identified four novel mutations in *CRB1* in a cohort of RP patients from the Chinese and Indian populations. Our data enlarges the *CRB1* mutation spectrums and may provide new target loci for RP diagnose and treatment.

Retinitis pigmentosa (RP) is a progressive photoreceptor dystrophy that primarily affects the rod photoreceptors whereas the function of the cone receptors is compromised as the disease progresses^1^. RP is the most common inherited retinal dystrophy, affecting approximately 1 in 3,500–5,000 individuals worldwide^2^. In RP patients, the phenotype can be limited to the eye (non-syndromic), or it can appear as part of a syndrome with extraocular disorders such as hearing loss, obesity, and neurologic diseases^3^. While RP is clinically and genetically heterogeneous, it can be inherited as an autosomal dominant (30–40%), autosomal recessive (50–60%), as well as X-linked (5–15%) trait^2^. Initial symptoms of RP include night blindness and a gradual constriction of peripheral visual field caused by rod photoreceptor lost. Secondary loss of cone photoreceptors may subsequently lead to impairment of central vision and ultimately legal blindness^1^. Due to its high heterogeneity and diversity of inheritance patterns, the clinical presentation of RP is much variable and the molecular diagnosis of syndromic and non-syndromic RP is very challenging.

At present, according to the RetNet database (https://sph.uth.edu/retnet/), more than 60 genes have been implicated in syndromic and non-syndromic forms of RP, which account for only about 60% of all RP cases. Autosomal recessive RP (arRP) is the most common form of RP worldwide and 36 genes/loci have been associated with autosomal recessive RP (RetNet) to date. Nevertheless, these mutations can only explain about 50–60% of RP cases and the heritability of 40% of the total RP cases worldwide remains unknown^4^.

Recently, the new next-generation sequencing (NGS) technology has been successfully used for the molecular diagnosis and gene identification of disease genes^5^,^6^. Coupled with DNA capture technology, next-generation sequencing (NGS) analysis enables rapid and efficient parallel sequencing of a large panel of RP disease genes^1^,^7^,^8^,^9^,^10^,^11^. Here, with the aim of investigating novel mutations in the RP disease, we performed whole exome sequencing in a Chinese family RP-2236, an Indian family RP-IC-90 and 100 sporadic Indian individuals with autosomal recessive RP (arRP).

## Materials and Methods

### Patients and Controls

Three RP patients and five unaffected family members from a four generation consanguineous marriage Chinese family RP-2236, One RP patients and two unaffected family members from an Indian family RP-IC-90, 100 sporadic Indian RP patients and 1000 control individuals with no history of retinal degeneration or other eye disease were collected from Hospital of the University of Electronic Science and Technology of China and Sichuan Provincial People’s Hospital and Aravind Eye Hospital of India. This research was carried out in accordance with the tenets of the Declaration of Helsinki and was approved by the Hospital of the University of Electronic Science and Technology of China and Sichuan Provincial People’s Hospital and Aravind Eye Hospital of India. All experiments were carried out in accordance with the approved protocol. Written informed consent was obtained from all subjects who participated in this study or from their legal guardians for minors.

### Clinical Diagnosis

A detailed patient history regarding disease onset, symptoms and progression and a family history were recorded. Complete ophthalmic examination of the patients were performed, including best corrected visual acuity (BCVA), fundus photography, multifocal electroretinography (mfERG), full field ERG (proband only) and fundus fluorescein angiography (FFA). Diagnosis of arRP was based on the presence of poor night vision, loss of peripheral visual field and abnormal fundus observations and ERG measurements. Clinical information about the Chinese family RP-2236 was listed in Table 1. All of the control individuals were underwent complete ophthalmic examination and were absent with any ocular fundus diseases.

### DNA Extraction

All genomic DNA samples were extracted from peripheral blood leukocytes of relative RP patients and control individuals using a blood DNA extraction kit according to the protocol provided by the manufacturer (TianGen, Beijing, China). DNA samples were stored at −20 °C until use. DNA integrity was evaluated by 1% agarose gel electrophoresis.

### Exome Sequencing and Data analysis

Exome sequencing was performed on DNA samples of patient IV:2 in the Chinese family RP-2236, patient II:1 in the Indian family RP-IC-90 and all of the 100 sporadic Indian patients by Axeq Technology Inc., Seoul, Korea. Each DNA samples was prepared by the Illumina protocols of Sure Select Target Enrichment System as described previously^12^.

Briefly, genomic DNA was fragmented by nebulization, the fragmented DNA was repaired, an ’A’ was ligated to the 3′ end, Illumina adapters were then ligated to the fragments, and the sample was size selected aiming for a 3502400 base pair product. The size-selected product was PCR amplified, and the final product was validated using the Agilent Bioanalyzer. Streptavidin beads were used to capture probes containing the targeted regions of interest; non-specific binding was then washed out. Then, the sequencing libraries were enriched for the desired target using the Illumina Exome Enrichment protocol and the enriched library validation for quality control analysis were performed by the Axeq Technology. For clustering and sequencing, genomic DNA Illumina Sure Select Target Enrichment System Capture Process was used to collect the protein coding regions of human genome DNA.

After exome sequencing, data analysis was performed. The reads were mapped against UCSC hg19 (http://genome.ucsc.edu/) by BWA (http://bio-bwa.sourceforge.net/). The SNPs and Indels are detected by SAMtools (http://samtools.sourceforge.net/). The detected variants were annotated and filtered based on four databases; i.e., NCBI CCDS (http://www.ncbi.nlm.nih.gov/CCDS/CcdsBrowse.cgi), RefSeq (http://www.ncbi.nlm.nih.-gov/RefSeq/), Ensembl (http:// www.ensembl.org), and Encode (http://genome.ucsc.edu/ENCODE). Four major steps were taken to explore the candidate variants: (i) all Functional_SNP/Indels were included through excluding variants within intergenic, intronic, and UTR regions and synonymous mutations; (ii) variants in the known RP gene (http://www.sph.uth.tmc.edu/retnet/) were included in downstream analysis; (iii) variants with high frequency in dbSNP137 (http://www.ncbi.nlm.nih.gov/projects/SNP/), 1000 Genome project (ftp://ftp.1000genomes.ebi.ac.uk/vol1/ftp), YH Database (http://yh.genomics.org.cn/), HapMap Project (ftp://ftp.ncbi.nlm.nih.gov/hapmap) and our in-house database, which was generated by whole exome sequencing technique in our laboratory using 1600 samples with no RP phenotype were excluded; (iv) homozygous variants and the compound heterozygous variants were included and the possible damaging impacts of each variant on protein structure/function were predicted by SIFT (http://sift.bii.astar.edu.sg/) and Polyphen2 (http://genetics.bwh. harvard.edu/pph2/).

### Mutations validation

The mutations in *CRB1* gene identified by the whole exome sequencing and data analysis were confirmed by direct Sanger sequencing in all of the family members, sporadic patients and 1000 normal controls. Sequencing data was used to determine whether the mutations were co-segregated with the disease in these families and whether the mutations were existed in the sporadic Indian patients or the normal control samples. Validation of the mutations was performed by Sanger sequencing on an ABI 3730XL Genetic Analyzer using the following primers: *CRB1*-c.2129-1G>C-F, 5′-CATGAAGTGCCCTTGTCCAA-3′; *CRB1*-c.2129-1G>C-R, 5′-ATGGAGAGGCTGATGGTGTC-3′. *CRB1*-p.R764C-F, TGAAGAGTATGTGGCAGGCA; *CRB1*-p.R764C-R, TCTTGCTTGTCAGGTAGGCC; *CRB1*-p.G1310C-F, TGGGTAGATAAGACTGTGCTGT; *CRB1*-p.G1310C-R, TCTTCCTGTTCACCCCACTC; *CRB1*-p.N629D-F, AGTGGCATTTCGTGGAGGTA; *CRB1*-p.N629D-R, CGTCCTCTG CTTTGACAAGG; *CRB1*-p.A593T-F, CTGGAGCTGCTAAGTGGCTA; *CRB1*-p.A593T -R, CACAGCCTGCCTTGACATTT.

## Results

### Patients and clinical information

**Figure 1:**
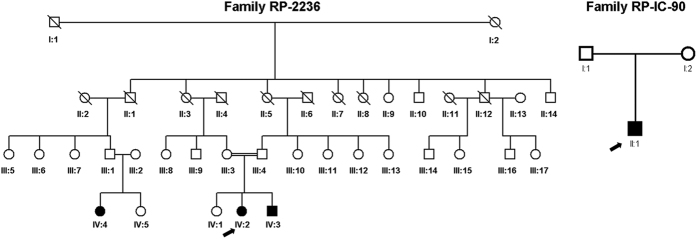
Pedigrees of the Chinese and Indian family with arRP. Arrow indicated the proband patient IV:2 in the Chinese family of RP-2236 (**A**) and the proband patient II:1 in the Indian family of RP-IC-90 (**B**). Solid symbol indicated affected individual while open symbols indicated unaffected individuals.

**Figure 2:**
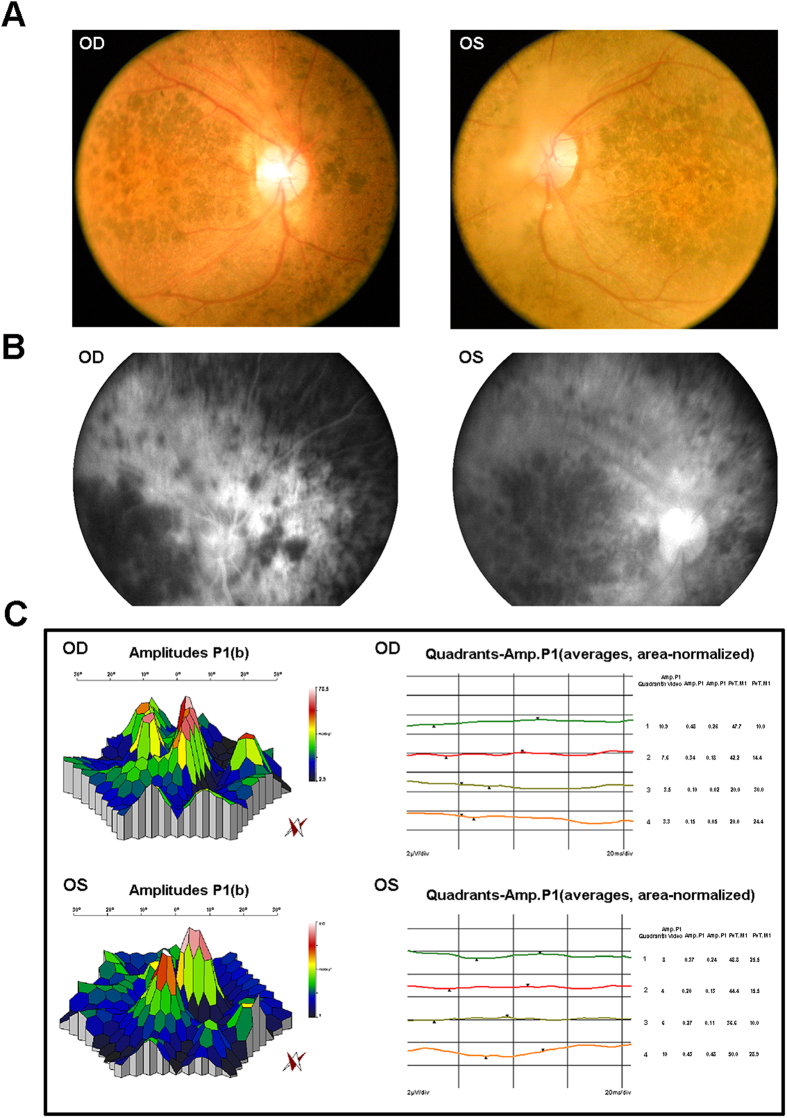
Representative clinical features of patient IV:2 in the Chinese family RP-2236. (**A**) Fundus photographs showed the loss of pigment epithelial with narrowed arterioles, pale optic disk and irregular pigment clumps with both peripheral retina and macula involved in both eyes. (**B**) FFA images showed extensive transmitted and blocked fluorescence due to loss of pigment epithelium with scattered pigment clumps. (**C**) mfERG records showed weak response under neither scotopic nor photopic condition, especially at the peripheral retina.

A Chinese family with three patients affected with retinitis pigmentosa, an Indian family with one patient affected with retinitis pigmentosa, 100 sporadic Indian RP patients and 1000 normal control were recruited in the study (Fig. 1). All of the RP patients presented typical RP clinical features, including early-onset increasing difficulty to adapt in the dark, gradually decreased vision acuity, and lost of peripheral and subsequent central visual field. The clinical examination of patient IV:2 in the Chinese family RP-2236 was shown in Fig. 2. Fundus examination showed the loss of pigment epithelial with narrowed arterioles, pale optic disk and irregular pigment clumps with both peripheral retina and macula involved in both eyes (Fig. 2A). The FFA showed extensive transmitted and blocked fluorescence due to loss of pigment epithelium with scattered pigment clumps (Fig. 2B). Both of the mfERG and full field ERG showed weak response under both scotopic and photopic condition, especially at the peripheral retina, indicating significant loss of function of both rods and cones (Fig. 2C and Supplementary Figure S1). Fundus photography of the other two patients in the family also showed similar changes to patient IV:2 (Supplementary Figure S2) The Indian RP patients and the normal controls recruited in this study also received complete ophthalmological examinations.

### Exome sequencing Analysis

We applied whole exome sequencing technique to analyze patient IV:2 in the Chinese family RP-2236, patient II:1 in the Indian family RP-IC-90 and 100 sporadic Indian RP patients and identified a large number of variants in each sample, including SNPs and Indels. The next-generation sequencing results of the familial samples and the 100 sporadic cases were shown in supplementary Table S1. For analysis, we firstly compared these variants with reported retina genes (https://sph.uth.edu/Retnet/), and then we found several variants of the known RP genes in these patients. By screening these variants though the dbSNP137, and 1000 Genome Project and our in-house database, described previously^12^, we exclude those variants with high frequency in the databases described above. By excluding heterozygous variants and synonymous mutations, we found a novel homozygous splicing mutation c.2129-1G>C in *CRB1* gene in patient IV:2 of the Chinese family RP-2236 and a homozygous missense mutation p.R664C in *CRB1* gene in patient II:1 of the Indian family RP-IC-90, respectively. The variants filtered by several database in the Chinese family RP-2236 and the Indian family RP-IC-90 were show in Table 2. In the 100 sporadic Indian patients, several mutations in the *CRB1* gene were identified, including a homozygous point mutation p.G1310C identified in Indian sporadic patient I-44, and compound heterozygous mutations p.N629D and p.A593T in Indian sporadic patient I-7.

After these steps, we identified five mutations in the RP gene *CRB1* in the Chinese and Indian populations. We checked these five mutations in the human gene mutation database (http://www.hgmd.org/) and the newly available ExAC database of 63,000 control exomes (http://exac.broadinstitute.org/). Except the p.R664C^13^ mutation, all other four mutations were not present in the two databases. Mutation locus, genotypes and prediction results by SIFT and PolyPhen2 of the family members and the sporadic patients were shown in Table 3.

### Mutation validation and Analysis

**Figure 3:**
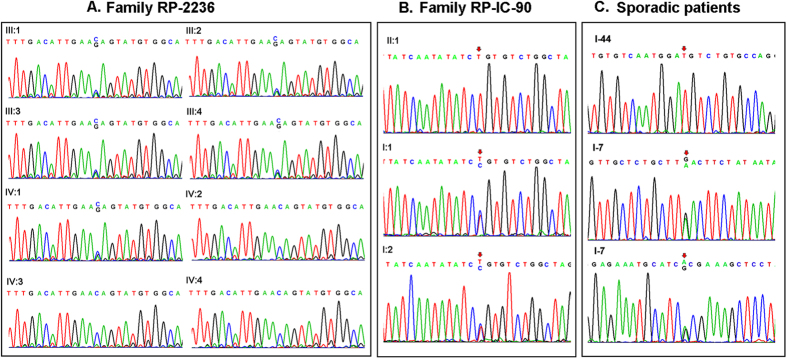
Mutation identification of *CRB1* gene in the Chinese family and the Indian family and the sporadic Indian patients with arRP. (**A**) Validation of the *CRB1* gene in the family RP-2236. Patients (IV:2, IV:3 and IV:4) harbored the homozygous splicing mutation c.2129-1G>C of the *CRB1* gene. The parents (III:1, III:2, III:3, III:4) and the healthy daughter (IV:1) were all unaffected carriers with the heterozygous c.2129-1G>C splicing mutation. (**B**) Validation of the *CRB1* gene in the family RP-IC-90. Patient II:1 harbored the homozygous point mutation p.R764C of the *CRB1* gene. The parents (I:1 and I:2) were unaffected carriers with the heterozygous p.R764C mutation. (**C**) Validation of the *CRB1* gene in the sporadic Indian patents. The patient I-44 harbored the homozygous mutation p.G1310C while the patient I-7 harbored the compound heterozygous mutations p.N629D and p.A593T.

Sanger sequencing results showed complete co-segregation of the mutations with the disease phenotype in the consanguineous Chinese family RP-2236 (Table 3). The homozygous splicing mutation c.2129-1G>C in the three affected patients (IV:2, IV:3 and IV:4) was confirmed (Fig. 3). The parents (III:1, III:2, III:3 and III:4) and the daughter (IV:1) were all unaffected carriers with a heterozygous mutation. The c.2129-1G>C splicing mutation in *CRB1* results in deletion of exon 7 and is likely to affect the *CRB1* protein function dramatically.

By Sanger sequencing, we conformed that patient II:1 of the Indian family RP-IC-90 was homozygous for the *CRB1* p.R764C mutation and his parents were an unaffected carriers with one heterozygous mutation (Fig. 3 and Table 3). In the previous report, the missense mutation p.R764C was revealed in retinitis pigmentosa 12, which introduces a substitution of Arginine to Cystine at the 764 amino acid of the *CRB1* protein and was predicted probably to be damaging to the protein function.

In the 100 sporadic RP patients, results of the whole exome sequencing were validated by Sanger sequencing analysis. Patient I-44 was identified with a homozygous missense mutation p.G1310C while patient I-7 was found with compound heterozygous mutations p.N629D and p.A593T. The Sanger sequencing results of the family members and the sporadic patients were shown in Fig. 3, while the genotypes of the family members and the sporadic patients were shown in Table 3.

We then screened these five variants in the 1000 ethnicity-matched control samples by Sanger sequencing. These mutation described above are absent in the 1000 control samples. These data, together with the clinical information, demonstrated that mutations c.2129-1G>C, p.R764C, p.G1310C, p.N629D and p.A593T in *CRB1* were responsible for recessive retinitis pigmentosa in these Chinese and Indian patients enlisted in this study. Importantly, four novel mutations c.2129-1G>C, p.G1310C, p.N629D and p.A593T were identified in *CRB1* gene in our RP patients.

## Discussion

*CRB1* mutations were reported to lead to severe congenital and early-onset retinal dystrophies (EORD) with different phenotypic manifestations, including Leber congenital amaurosis (LCA), retinitis pigmentosa and cone-rod dystrophies, depending on the amount of residual *CRB1* activity and the genetic background^13^,^14^,^15^. LCA is the most severe nonsyndromic retinal dystrophy, characterized by blindness or severe visual impairment from birth^16^,^17^,^18^,^19^,^20^. In contrast, RP is considered a milder and more heterogeneous disorder, with a late age of onset. It is characterized by night blindness followed by gradual loss of peripheral vision, progressive degeneration of photoreceptors, and eventually leads to visual impairment of variable severity that in rare cases can result in complete blindness^3^,^4^,^21^,^22^. In this study, we identified mutations in *CRB1* in the Chinese and Indian populations with the autosomal recessive retinitis pigmentosa using the whole exome sequencing technology.

*CRB1* is a human homologue of the *Drosophila melanogaster* gene coding for protein crumbs (*crb*) and it is expressed in the brain and the inner segments of mammalian photoreceptors^13^,^16^,^19^,^23^,^24^,^25^,^26^,^27^. *CRB1* gene, located in 1q31, consists of 12 exons and exhibits alternative splicing at the 3′end, yielding four known isoforms, the longest of which consists of 1406 amino acids and contains 19 epidermal growth factor (EGF)–like domains, three laminin A globular (AG)–like domains, and a signal peptide in the extracellular region^23^,^24^. In addition, there is a C-terminal transmembrane domain. The cytoplasmic region contains a conserved FERM binding domain, involved in localizing proteins to the plasma membrane, and a PDZ binding motif (PBM). The Crumbs protein has been implicated in mechanisms that control cell to cell adhesion, intracellular communication, apicobasal cell polarity, and photoreceptor morphogenesis, which is critical for proper development and function in epithelial cells and photoreceptors^16^,^19^,^23^,^24^,^25^,^26^,^28^.

To date, over 150 different mutations have been identified in *CRB1* and most of the mutations occurs in exon 7 (27%) and exon 9 (41%), which encodes the second and the third laminin AG-like domains, respectively^29^. And the prevalence of *CRB1* mutations varies widely depending on the clinical phenotype and the cohort studied, ranging between 7% in EORD patients^30^, 10% in LCA patients^18^, 31% in RP patients with Coats-like exudative vasculopathy and 66% in RP patients with PPRPE^31^. Meanwhile different regions and populations have different prevalence of *CRB1* mutations. In the Israeli and Palestinian populations, *CRB1* mutations are relatively frequent causes of 10% of early-onset retinal degeneration^32^, while in the Spanish population *CRB1* have been shown as the main mutated gene in 17% of LCA patients^33^, but it seems to explain only about 2% of EORP^34^. Otherwise, the prevalence of *CRB1* mutations in the Chinese population with retinal disease was unknown for the lacking of enough samples and this will be a subject of further studies.

**Figure 4:**
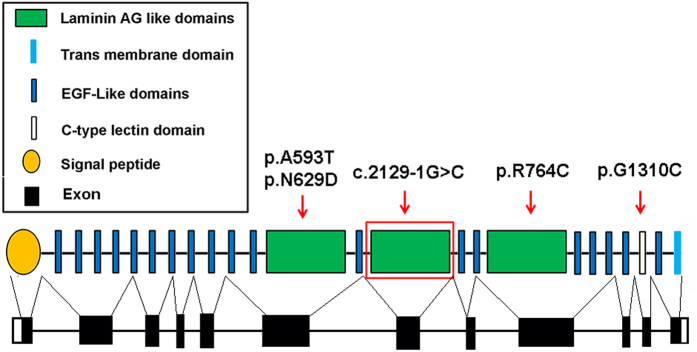
Schematic representation of the crumbs homolog 1 gene structure. The red arrow indicated the mutations reported in our study. The homozygous splicing mutation c.2129-1G>C in the *CRB1* gene damaged the second laminin AG-like domain which located in the exon 7; The reported mutation p.R764C located in the exon 9 and damaged the third laminin AG-like domain; The mutation of p.G1310C located in the exon 11 and affected the function of the C-type lectin domain. The compound heterozygous mutation p.N629D and p.A593T both located in the exon 6 and affected the function of the first laminin AG-like domain.

In the present study, our whole exome sequencing and data analysis in an arRP Chinese family revealed a novel homozygous splicing mutation c.2129-1G>C in *CRB1* according to GenBank accession number NM_001193640.1. This homozygous splicing mutation results in deletion of exon 7, which encodes the second laminin AG-like domain. As previously reported, these laminin AG-like domains are conserved from flies to mammals^35^ and are predicted to affect protein to protein interactions, calcium binding, or protein folding. Even one amino acid change results in loss function of the laminin AG-like domain. Therefore, the splicing mutation c.2129-1G>C in *CRB1* may damage the laminin AG-like domain function more severely than missense mutations. The p.R764C mutation in *CRB1* was previously reported in retinitis pigmentasa 12 in the Caucasian population in 1999^13^, our result confirmed that this mutation caused recessive retinitis pigmentosa in the Indian population. The homozygous point mutation of p.G1310C introduces a substitution of Glycine to Cystine in the exon 11 of the *CRB1* protein and is predicted probably to be damaging to the protein function through the C-type lectin domain (SIFT score is 0.002 and PolyPhen2 scores close to 1.0), thus causing retinitis pigmentosa in the Indian RP patient. The compound heterozygous mutations p.N629D and p.A593T were located in exon 6 of the *CRB1* gene in the Indian population and likely affect the function of the first laminin AG-like domain of *CRB1* protein dramatically. Topological organization of *CRB1* and the five mutations reported in our study was shown in Fig. 4. These mutations described above were absent from public databases such as 1000 genomes or Exome Variant Server, excluding them as common polymorphisms. However, the effect of these mutations on the function of *CRB1* has not yet to be determined. In order to better understand RP pathogenesis, functional studied are necessary to confirm the roles of these mutations in *CRB1* gene function and the underlying molecular mechanisms.

Whole exome sequencing (WES) technology is a powerful method to identify potential genetic variants in inherited retinal disease more efficiently and accurately. Moreover, recent improvement in WES technologies and their progressive implementation in the clinical diagnosis will help to improve the molecular diagnosis retinal dystrophies. In conclusion, our present study reveals five mutations in *CRB1* gene in Chinese and Indian populations with retinitis pigmentosa by the whole exome sequencing. Our data expand the *CRB1* mutation spectrums and may provide new target loci for RP diagnose and treatment.

## Additional Information

**How to cite this article**: Yang, Y. *et al*. Whole exome sequencing identified novel *CRB1* mutations in Chinese and Indian population with autosomal recessive retinitis pigmentosa. *Sci. Rep.* **6**, 33681; doi: 10.1038/srep33681 (2016).

## Supplementary Material

Supplementary Information

## Figures and Tables

**Table 1:** Clinical information of members in the Chinese family RP-2236.

Family Member	Age	Gender	Relation–ship	Clinical Information				
				Disease onset(years old)	Visual Acuity(OD/OS)	Fundusexamination	mfERG	FFA
III1	46	M	Father	/	1.2/1.2	Normal	Normal	Normal
III2	43	F	Mother	/	1.0/1.0	Normal	Normal	Normal
III3	48	M	Mother	/	1.2/1.0	Normal	Normal	Normal
III4	45	F	Father	/	1.0/1.2	Normal	Normal	Normal
IV:1	22	F	Daughter	/	1.2/1.2	Normal	Normal	Normal
IV:2	18	F	Daughter	2	0.05/0.05	Loss of pigment epithelium	Weak response	Extensive transmitted and blocked fluorescence
IV:3	16	M	Son	1	0.05/0.05	Loss of pigment epithelium	Weak response	Extensive transmitted and blocked fluorescence
IV:4	19	F	Daughter	1.5	0.05/0.05	Loss of pigment epithelium	Weak response	Extensive transmitted and blocked fluorescence

M, male; F, female; /, no eye disease; FFA, fundus fluorescein angiography; mfERG, multifocal electroretinography.

**Table 2:** Number of candidate SNP/Indels in patient IV:2 of the consanguineous Chinese family RP-2236 and patient II:1 of the Indian family RP-IC-90 filtered against several public variation databases and the in-house data.

	Feature_SNPs and Indels inpatient IV:2 of family RP-2236	Feature_SNPs and Indels inpatient II:1 of family RP-IC-90
Total_SNPs/Indels	65170/8305	66320/7901
Functional_SNP/Indels	366/120	387/109
Filtered_known gene	60	42
Filtered_DBsnp137common/indel;Filtered_DBsnp/indel_1000gene(2011); MAF <0.05	5	3
Filtered in House Data	3	2
homozygous	1	1
Filtered synonymous mutation	1	1

**Table 3:** Genotypes of the family members and the sporadic patients.

		Phenotype	Nucleotide change	Effect	Genotype	SIFT, Polyphen2	Original reports described
Family RP-2236	III1	/	c.2129-1G>C	Splicing	heterozygous	N/A, N/A	N/A
	III2	/	c.2129-1G>C	Splicing	heterozygous	N/A, N/A	N/A
	III3	/	c.2129-1G>C	Splicing	heterozygous	N/A, N/A	N/A
	III4	/	c.2129-1G>C	Splicing	heterozygous	N/A, N/A	N/A
	IV:1	/	c.2129-1G>C	Splicing	heterozygous	N/A, N/A	N/A
	IV:2	RP	c.2129-1G>C	Splicing	homozygous	N/A, N/A	N/A
	IV:3	RP	c.2129-1G>C	Splicing	homozygous	N/A, N/A	N/A
	IV:4	RP	c.2129-1G>C	Splicing	homozygous	N/A, N/A	N/A
Family RP-IC-90	I:1	/	c.C2290T	p.R764C	heterozygous	Damaging, Probably damaging	den Hollander AI
	I:2	/	c.C2290T	p.R764C	heterozygous	Damaging, Probably damaging	den Hollander AI
	II:1	RP	c.C2290T	p.R764C	homozygous	Damaging, Probably damaging	den Hollander AI
Sporadic patients	I-44	RP	c.G3928T	p.G1310C	homozygous	Damaging, Probably damaging	N/A
	I-7	RP	c.A1885G	p.N629D	heterozygous	Damaging, Probably damaging	N/A
			c.G1777A	p.A593T	heterozygous	Damaging, Probably damaging	N/A

/, no eye disease; N/A, Not available.
