# Supplementary material for: Whole exome sequencing identified novel CRB1 mutations in Chinese and Indian populations with autosomal recessive retinitis pigmentosa
Source: Sci Rep. 2016 Sep 27;6:33681. doi: 10.1038/srep33681 (PMC5037368; doi:10.1038/srep33681)
Supplement: Supplementary Information [file srep33681-s1.pdf]

Whole exome sequencing identified novel *CRB1* mutations in Chinese and Indian population with autosomal recessive retinitis pigmentosa

Yin Yang [1,2*], Yeming Yang[1,3,4,6*], Lulin Huang[1,3], Yaru Zhai[1], Jie Li[2], Zhilin Jiang[1,3], Bo Gong[1,3], Hao Fang[1,3], Ramasamy Kim[5], Zhenglin Yang[1,3,6], Periasamy Sundaresan[7], Xianjun Zhu[1,3,4,6,8#], Yu Zhou[1,3,6,8#]

* Contributed equally to this work

Supplementary information file

Supplementary information file includes one table (table S1) and two figures (figure S1 and figure S2).

Table S1

The next-generation sequencing results of the familial samples and the 100 unrelated cases.

	total reads that pass QC	Initial mappable reads	On-target reads	% Coverage of target regions(more than 10×)	Mean read depth of target regions
IV:2 in RP-2236	46,130,930	45,962,518	34,249,841	94.6%	57.0
II:1 in RP-IC-90	53,474,296	53,303,980	40,791,416	96.3%	68.6
100 sporadic samples(average)	49,479,221±9819444.07	49,295,539±9779248.38	37,721,369±4228047.62	95.2%±1.32%	62.3±5.32

Figure Legends

Figure S1. The full field ERG of patient IV:2. The full field ERG test of patient IV:2 showed almost no response under the scotopic condition and weak response under the photopic condition. The Incubation period of b wave prolonged and its amplitude decreased. This test indicated significant loss of function of both rods and cones.

Figure S2. Fundus photographs of other patients. (A) and (B) Fundus photography of patient IV:3 showed pale optic papilla, thinning of retinal vessels and pigmentation all over the retina in each eye. (C) and (D) Fundus photography of patient IV:4 showed unclear boundary of optic papilla, thinning of retinal vessels as well as pigment disorder and deposition in both eyes.

Fig S1

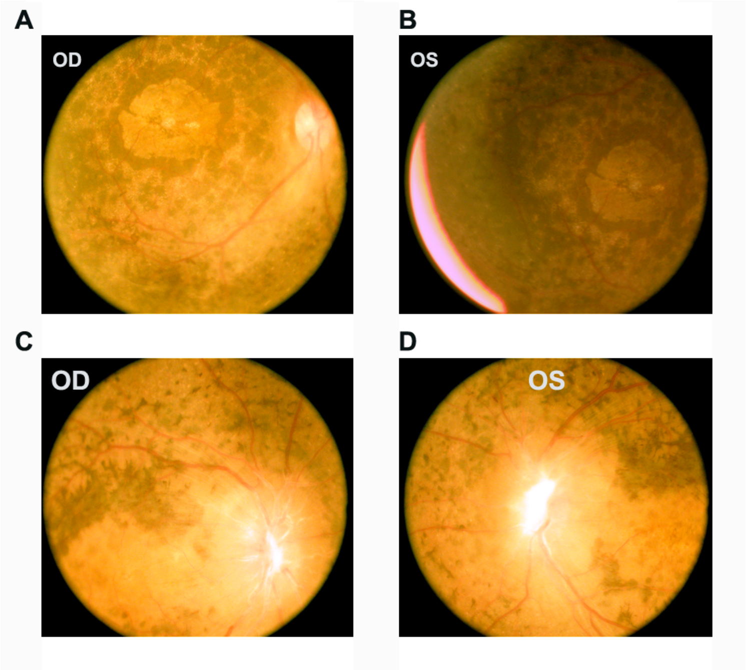

Fig S2

Diagnosis:

1_Scotopic 0.01 ERG (GF)

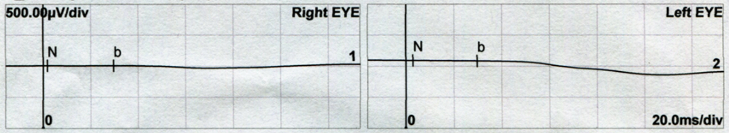

Normals	74-90	289μV-437μV
Channel	b [ms]	b-wave
1 R-1	29.9 (!)	1.13μV (!)
2 L-2	29.9 (!)	7.46μV (!)

2_Scotopic 3.0 ERG (GF)

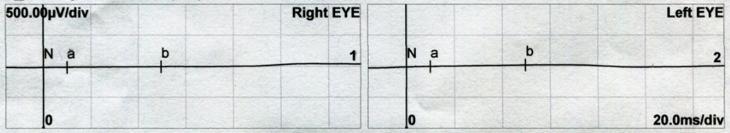

Normals	16-23	43-54	254μV-411μV	515μV-694μV	1.50-2.60
Channel	a [ms]	b [ms]	a-wave	b-wave	b/a
1 R-1	10.0 (!)	49.9	4.38μV (!)	10.4μV (!)	2.37
2 L-2	10.0 (!)	49.9	9.30μV (!)	25.8μV (!)	2.77

3_Scotopic 10.0 ERG (GF)

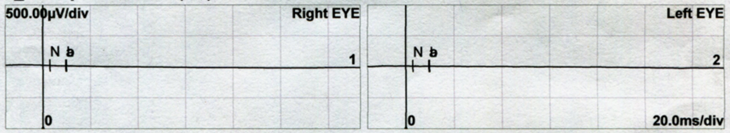

Channel	a [ms]	b [ms]	a-wave	b-wave
1 R-1	9.7	10.0	4.59μV	150nV
2 L-2	9.7	10.0	512nV	8.40nV

4_Scotopic 3.0 Oscillatory Potential ERG (GF)

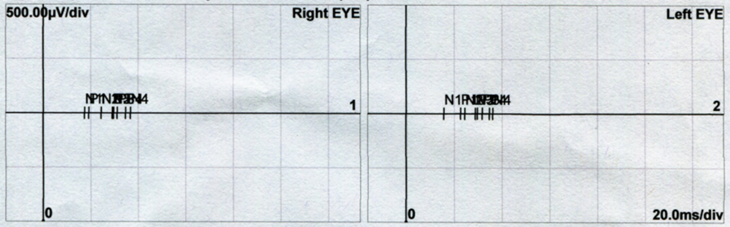

Normals	-	-	21-26	-	-	-	-		91.0μV-170μV			
Channel	P1 [ms]	N2 [ms]	P2 [ms]	N3 [ms]	P3 [ms]	N4 [ms]	P4 [ms]	OS1	OS2	OS3	OS4	TotalOS
1 R-1	19.4	24.7	29.9 (!)	29.4	31.4	37.0	34.9	32.7nV	130nV (!)	96.2nV	181nV	440nV
2 L-2	22.9	24.7	29.9 (!)	29.1	32.0	36.4	34.9	593nV	273nV (!)	359nV	337nV	1.56μV

5_Photopic 3.0 ERG (GF)

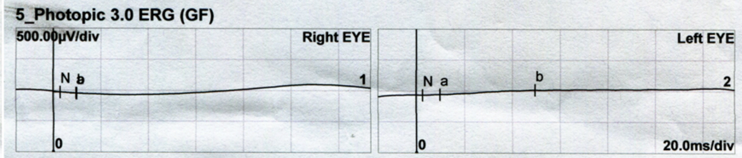

Normals	15-17	27-34	27.0μV-92.0μV	133μV-220μV
Channel	a [ms]	b [ms]	a-wave	b-wave
1 R-1	9.7 (!)	10.0 (!)	17.9μV (!)	523nV (!)
2 L-2	10.0 (!)	49.9 (!)	10.0μV (!)	76.1μV (!)

6_Photopic 3.0 Flicker 30Hz ERG (GF)

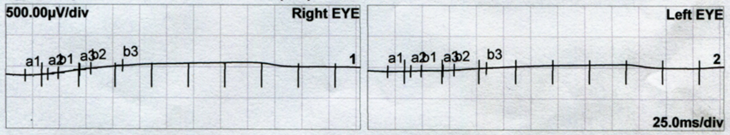

Normals	-	-	58-64	-	-		75.0μV-205μV	
Channel	b1 [ms]	a2 [ms]	b2 [ms]	a3 [ms]	b3 [ms]	a1-b1	a2-b2	a3-b3
1 R-1	49.7	40.0	80.1 (!)	69.7	109.8	41.4μV	95.0μV	72.5μV
2 L-2	49.7	40.0	80.1 (!)	69.7	109.8	14.1μV	22.0μV (!)	41.9μV
